# Twirling and Spontaneous Symmetry Breaking of Domain Wall Networks in Lattice-Reconstructed Heterostructures of Two-Dimensional Materials

**DOI:** 10.1021/acs.nanolett.3c01896

**Published:** 2023-10-02

**Authors:** Mikhail A. Kaliteevski, Vladimir Enaldiev, Vladimir I. Fal’ko

**Affiliations:** † National Graphene Institute, University of Manchester, Booth Street East, Manchester M13 9PL, United Kingdom; ‡ Department of Physics and Astronomy, University of Manchester, Manchester M13 9PL, United Kingdom; § Henry Royce Institute for Advanced Materials, Manchester M13 9PL, U.K.

**Keywords:** 2D materials, dislocation, phase transitions, twistronics, spontaneous symmetry breaking

## Abstract

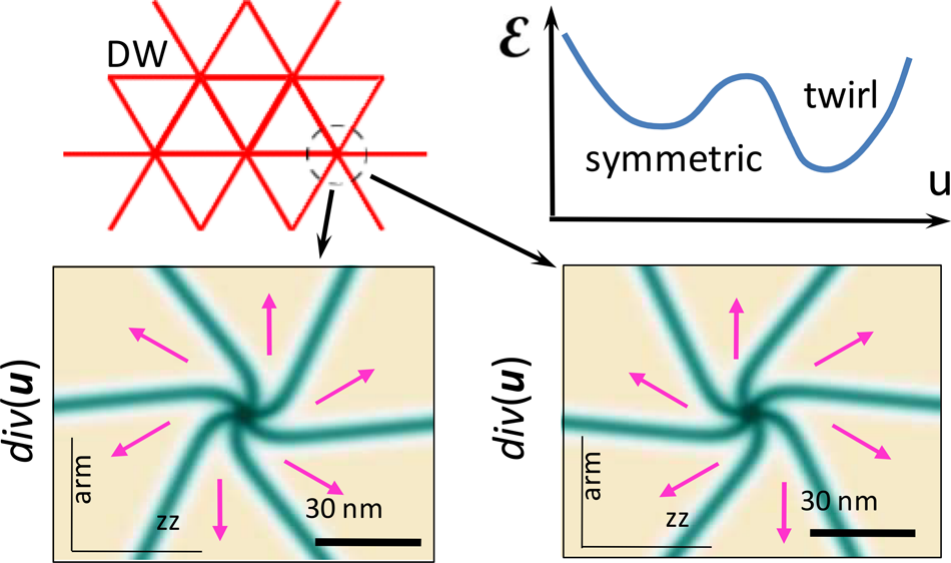

Lattice relaxation in twistronic bilayers with close lattice parameters and almost perfect crystallographic alignment of the layers results in the transformation of the moiré pattern into a sequence of preferential stacking domains and domain wall networks. Here, we show that reconstructed moiré superlattices of the perfectly aligned heterobilayers of same chalcogen transition metal dichalcogenides have broken-symmetry structures featuring twisted nodes (“twirls”) of domain wall networks. The analysis of twist-angle dependence of strain characteristics for the broken-symmetry structures shows that the formation of twirl reduces the amount of hydrostatic strain around the nodes, potentially weakening their influence on the band edge energies of electrons and holes.

This study addresses a detailed analysis of domain wall networks (DWNs) that form in long-period moiré patterns characteristic of highly aligned heterostructures of same chalcogen transition metal dichalcogenides MoX_2_/WX_2_ (TMDs with X = S or Se). As same chalcogen TMDs have very close lattice constants, moiré patterns at their interface have long periods offering sufficient space for the formation of preferential stacking areas (domains). In other words, the energy gain due to better adhesion can surmount the cost of intralayer strain in each of the constituent crystals. The reconstruction of small-angle twisted bilayers into an array of domains^1^ has been observed^2−6^ in both MoS_2_/WS_2_ and MoSe_2_/WSe_2_ heterostructures. The observed^2−6^ and theoretically modeled^1,7,8^ structures feature hexagonal DWNs for the antiparallel (AP) orientation and triangular DWNs for the parallel (P) orientation of TMD unit cells.

Moreover, curling of DW near network nodes has been suggested for small-angle twisted bilayer graphene^9^ and some TMD homobilayers,^10^ accompanied by breaking both rotational and translational symmetries of DWNs.

Here, we show that lattice relaxation in P/AP-MoX_2_/WX_2_ (X = S or Se) bilayers in the twist-angle limit of θ = 0° undergoes symmetry breaking of the domain wall patterns that results in the formation of twirled structures of DWN nodes (see Figure 1 for P-MoX_2_/WX_2_ bilayers).

**Figure 1:**
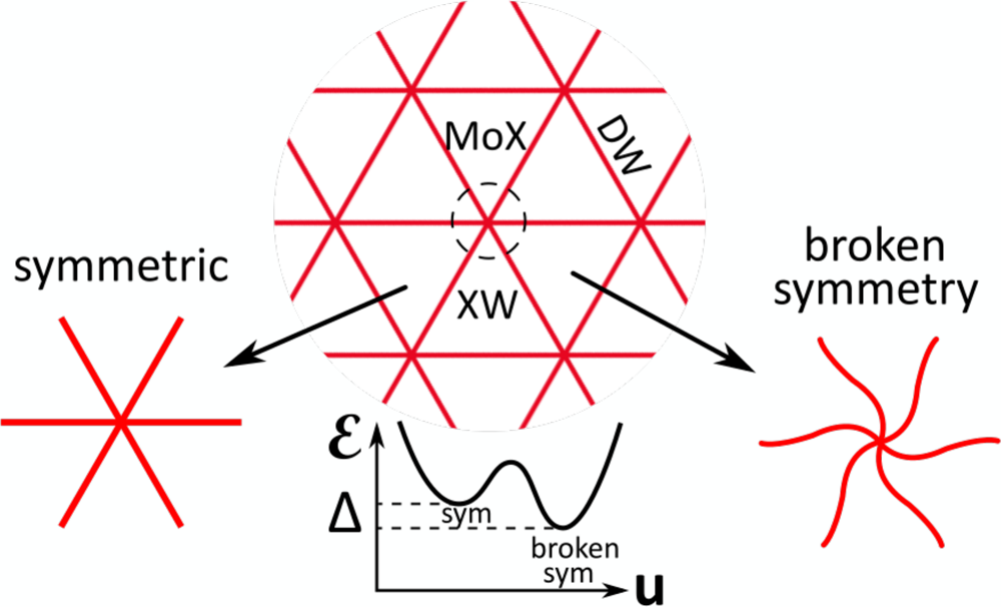
Symmetric and broken-symmetry (twirl) structures of DWN in aligned P-MoX_2_/WX_2_ bilayers. The bottom plot shows the energy gain (Δ) from the formation of twirls.

In contrast to the deformations of DWN nodes in homobilayers mentioned above,^9,10^ the twirling in MoX_2_/WX_2_ heterobilayers happens for zero twist angle between the constituent layers, spontaneously choosing the left or right twirling direction across entire DWNs and preserving the initial periodicity of the moiré superlattice set by the incommensurability between MoX_2_ and WX_2_ lattice constants. As a result, the predicted twirling represents a true symmetry breaking of the entire moiré superlattice toward left-handed or right-handed structure. This effect stems from the hydrostatic strain component determined by a small lattice mismatch of the two constituent two-dimensional (2D) crystals. Via adjustment of the lattice constants of MoX_2_ and WX_2_ inside the domains, each monolayer compression/expansion is inflicted on domain walls creating hot spots of hydrostatic strain in the DWN nodes with XX (chalcogen over chalcogen) stacking.^11^ The excessive energy cost of a large hydrostatic strain around XX nodes (as compared to shear deformations dominating DWN structure in homobilayers) is, then, negotiated by left- or right-handed twirling of the domain walls, leading to the broken-symmetry configuration sketched in Figure 1.

## Methods

To study lattice relaxation in MoX_2_/WX_2_ bilayers, we use a multiscale modeling approach developed in refs (1) and (12). This combines mesoscale elasticity with an analytically interpolated description of the adhesion energy of two layers, computed using density functional theory (DFT). The latter is represented below upon taking into account interlayer distance relaxation (see the Supporting Information):
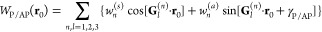
1where **r**_0_ is a locally defined in-plane vector determining the stacking arrangement between layers [**r**_0_ = 0 for XX stacking,  for XW/2H stacking, and  for MoX/MoW stacking in P/AP heterostructures], phases γ_P_ = π/2 and γ_AP_ = 0 account for **r**_0_ → −**r**_0_ symmetry of the adhesion energy in P and AP bilayers,^1^ and **G**_1,2,3_^(1,2,3)^ are three sets of the shortest reciprocal lattice vectors [|**G**_1,2,3_^(1)^| = *G*=**4π**/*a√3*, , and |**G**_1,2,3_^(3)^| = 2*G*] of a commensurate MoX_2_/WX_2_ heterostructure, related by 120° rotations within each set. The values of parameters *w*_1,2,3_^(*s,a*)^ established using DFT^1,12^ are listed in Table 1. Note that for each **r**_0_ the values of *W*_P/AP_(**r**_0_) correspond to the energetically optimal interlayer distance.^1^

**Table 1:** Adhesion^1^ and Elastic^13,14^ Energy Parameters (in electronvolts per square nanometer) Used in eq 3

X	*w*_1_^(*s*)^	*w*_1_^(*a*)^	*w*_2_^(*s*)^	*w*_2_^(*a*)^	*w*_3_^(*s*)^	*w*_3_^(*a*)^
S, AP	0.1415	0.0269	–0.0338	0	–0.0166	–0.0030
S, P	0.1559	0	–0.0398	0	–0.0199	0
Se, AP	0.1128	0.0256	–0.0201	0	–0.0098	–0.0024
Se, P	0.1284	0	–0.0248	0	–0.0124	0

X	λ_W_	λ_Mo_	μ_W_	μ_Mo_
S	328.2	520.1	453.2	443.0
Se	185.5	264.3	302.6	310.3

Next, we combine eq 1 with elasticity theory by substituting local stacking in the form
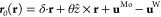
2where δ ≈ 0.2% for MoS_2_/WS_2_ bilayers and δ ≈ 0.4% for MoSe_2_/WSe_2_ and **u**^Mo^(**r**) and **u**^W^(**r**) are in-plane displacement fields describing lattice relaxation in the MoX_2_ and WX_2_ layers, respectively. Then, we minimize the total energy of the moiré superlattice
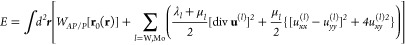
3with respect to displacement fields **u**^W^ and **u**^Mo^. Here, λ_Mo,W_ and μ_Mo,W_ are elastic moduli of MoX_2_ and WX_2_ monolayers, respectively (see Table 1), and *u*_*ij*_^*l*^ = (∂_*j*_*u*_*i*_^*l*^ + ∂_*i*_*u*_*j*_^*l*^)/2 are components of strain tensors.

To find optimal distributions of displacement fields, **u**^W^ and **u**^Mo^, we solve a system of Lagrange–Euler equations with periodic boundary conditions, implemented via the finite difference method. In particular, we chose a rectangular supercell with  and  sides ( is the moiré superlattice period, and *a* is the averaged monolayer lattice parameter), setting the following boundary conditions: , at 0 ≤ ±*y′* ≤ /2) = **u**^Mo/W^(*x′*, −/2). Here, prime superscripts indicate that the *x′Oy′* reference frame was rotated by an angle  with respect to the fixed frame for which *Ox* and *Oy* axes are along the zigzag and armchair directions in the crystal, respectively. Such a rotation allows us to fix boundary conditions (note that the Lagrange–Euler equations themselves are rotational-invariant).

In the numerical analysis, we use a sufficiently dense grid (one point per nanometer) to provide convergence of the computed displacement fields. This requires solving ∼30 000 coupled nonlinear equations. This solution is obtained in an annealing-type computational scheme. First, we choose the starting point as **u**^Mo/W^ = 0 and scale down the adhesion energy parameters *w*_1,2,3_^(*s*,*a*)^ by a small factor η = 10^–3^, finding a slightly relaxed structure. Then, we gradually increase this factor, up to 1 (at the final step), using solutions obtained at the previous iterations as starting points at each next step.

### Twirls in P-MoX_2_/WX_2_ Heterostructures

**Figure 2:**
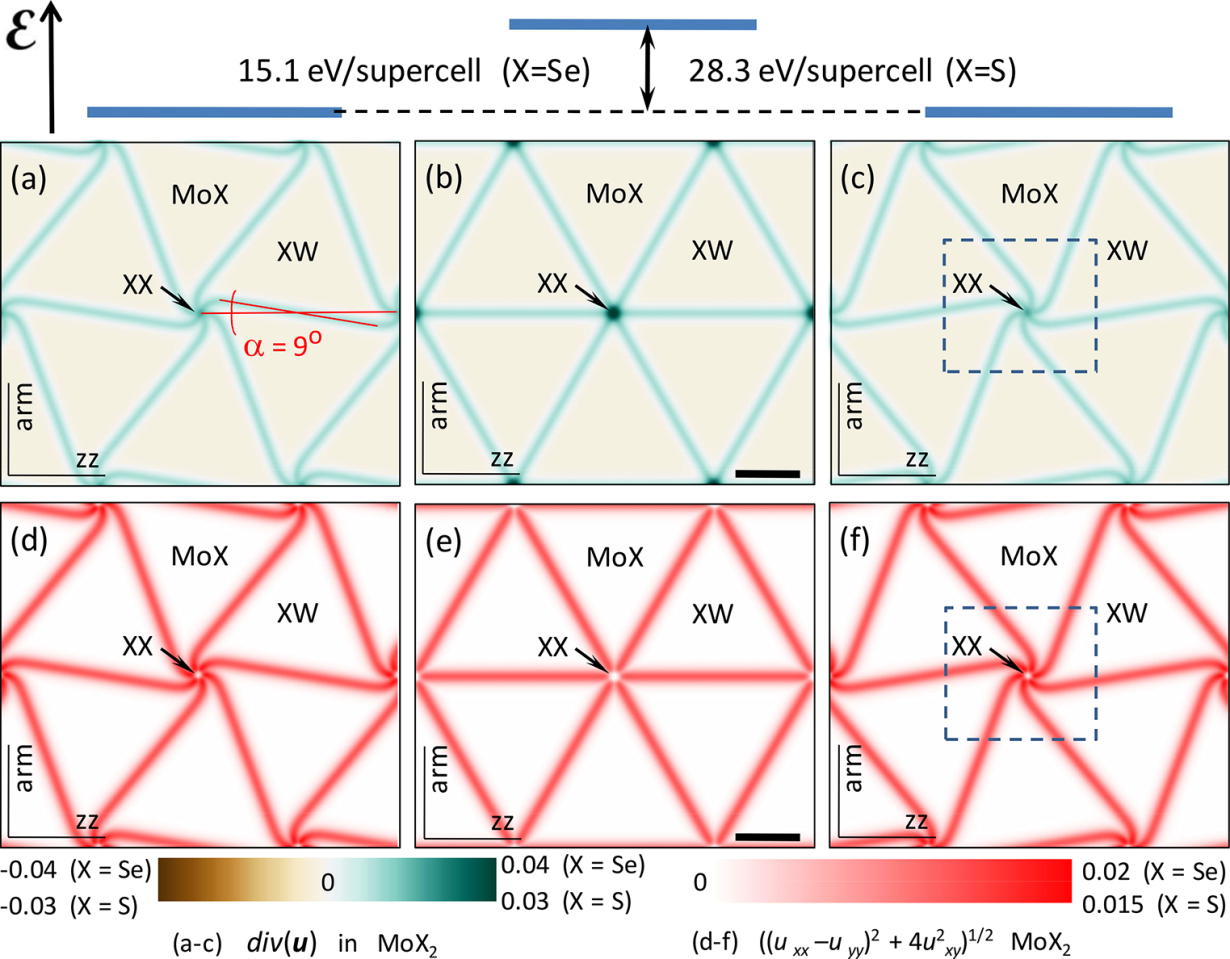
Maps of (a–c) hydrostatic and (d–f) shear components of the strain tensor in principal axes for (b) symmetric and (a and c) twirled structures of the reconstructed moiré superlattice in aligned P-MoX_2_/WX_2_ bilayers. The top sketch shows the energy gain from the formation of twirled structures. The scale bars are 30 and 45 nm for X = Se and X = S, respectively. Dashed rectangles in panels c and f show the area displayed in Figure 3.

**Figure 3:**
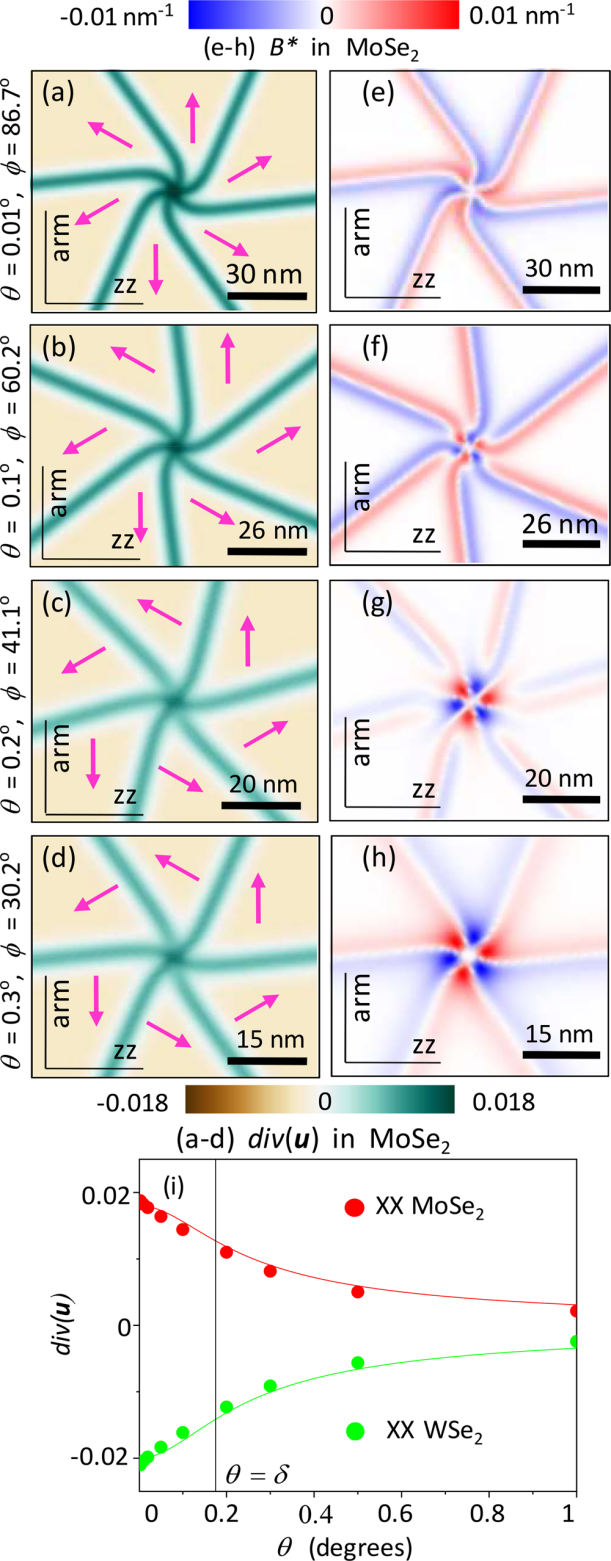
Twirl in P-MoSe_2_/WSe_2_ for θ = 0.01°, 0.1°, 0.2°, and 0.3°. (a–d) Maps of div**u**_Mo_. The arrows indicate the direction of stacking vector **r**_0_ () inside domains. (e–h) B* = 2∂_*x*_*u*_*xy*_^Mo^ + ∂_*y*_(*u*_*xx*_^Mo^ – *u*_*yy*_^Mo^). (i) Values of div **u**_Mo,W_ at the twirl center for various twist angles.

Lattice relaxation in P-MoX_2_/WX_2_ heterostructures results in the formation of DWNs separating triangular domains with MoX and XW stackings, similar to those found in 3R-TMD polytypes^2,3^ (see Figure 2). Minimizing functional 3, we find the following three competing distributions of strain. One of them (symmetric), shown in the middle panels, is characterized by straight edge dislocation lines linking XX stacking nodes of DWN. The other two (left and right panels) are twirled (broken-symmetry) structures of DWN nodes with a left/right-handed twist. The computed total energies per supercell, listed in Table 2, show that the energy of the twirled DWN structure is lower than that of the symmetric one. Note that left/right-handed twirls have the same energy, suggesting that the DWN in a perfectly aligned heterostructure (θ = 0°) undergoes a spontaneous symmetry breaking into a left- or right-handed twirled state across the entire DWN. We tested the persistence of only one (either left- or right-handed) orientation of twirls by computing the relaxed structures across multiple enlarged supercell periods (see Figure S7a–c). Twisting the heterobilayer to a small θ of >0° promotes right-handed twirling, with the energy difference between the right- and left-handed structures described in Figure S6. This distinguishes twirls in Figure 3 and Figure S7 from moiré pattern asymmetry in TMD homobilayers; random twisting of DWN nodes across the network was discussed in ref (10). We also note the DWN computation for homobilayers performed using the same approach as described in Methods and implemented across multiple moiré supercells does not produce twirled structures (see Figure S7d–f).

**Table 2:** Adhesion, Elastic, and Total Energies (in electronvolts per supercell) Computed for Symmetric (sym) and Twirled (twirl) Moiré Superlattice Structures and Their Total Energy Differences, Δ, in Aligned P-MoX_2_/WX_2_ Bilayers

X	structure	elastic	adhesion	total	Δ
Se	sym	223.01	–2349.68	–2126.67	15.12
	twirl	208.95	–2350.74	–2141.79	
S	sym	349.92	–5321.11	–4971.19	28.26
	twirl	323.33	–5322.78	–4999.45	

Values of adhesive and elastic energies compared in Table 2 also suggest that the dominant energy gain for the twirled structures comes from decreasing the elastic energy. The latter is determined by hydrostatic strain, div **u**, and shear deformations, characterized by vector **A** = (*u*_*xx*_ – *u*_*yy*_, −2*u*_*xy*_). Their distribution across DWNs is shown Figure 2, in the form of color maps for div **u** and |**A**|. Here, the difference between symmetric and twirled structures is such that the hydrostatic strain component, concentrated around nodes, is higher for the symmetric one. Because the energy costs of the hydrostatic strain (∝λ + μ) are higher than those of shear strain (∝μ), the twirled structures emerge as energetically favorable.

In Figure 3, we analyze the dependence of div **u** and **A** on the twist angle. The data shown in Figure 3i indicate that the maximal value of div **u** at the hot spot of strain decreases with twist, which also explains why twirling is weaker for larger θ values. Another deformation field characteristic that is interesting to consider is B* = [rot **A**]_*z*_ = 2∂_*x*_*u*_*xy*_^Mo(W)^ + ∂_*y*_[*u*_*xx*_^Mo(W)^ – *u*_*yy*_^Mo(W)^]. This characteristic determines the size of piezoelectric charges, generated by inhomogeneous strain in each layer, and pseudomagnetic field that would be experienced by charge carriers at the K-valley band edge in the heterostructure.^1,12^ Note that in P-heterostructures piezocharges (∝*e*_11_B*) have opposite signs in MoX_2_ and WX_2_ layers, as *B*_Mo_^*^ ≈ −*B*_W_^*^, and piezocoefficients have the same signs, *e*_11_^Mo^ ≈ *e*_11_^W^. For the displayed range of angles, 0° ≤ θ ≲ 0.4°, distributions of B* show the change of sign and zero value in the middle of the domain wall. For larger twist angles θ ≫ δ, piezocharge and pseudomagnetic field distributions take the form of those established earlier for homobilayers.^1^

### Twirls in AP-MoX_2_/WX_2_ Heterostructures

**Figure 4:**
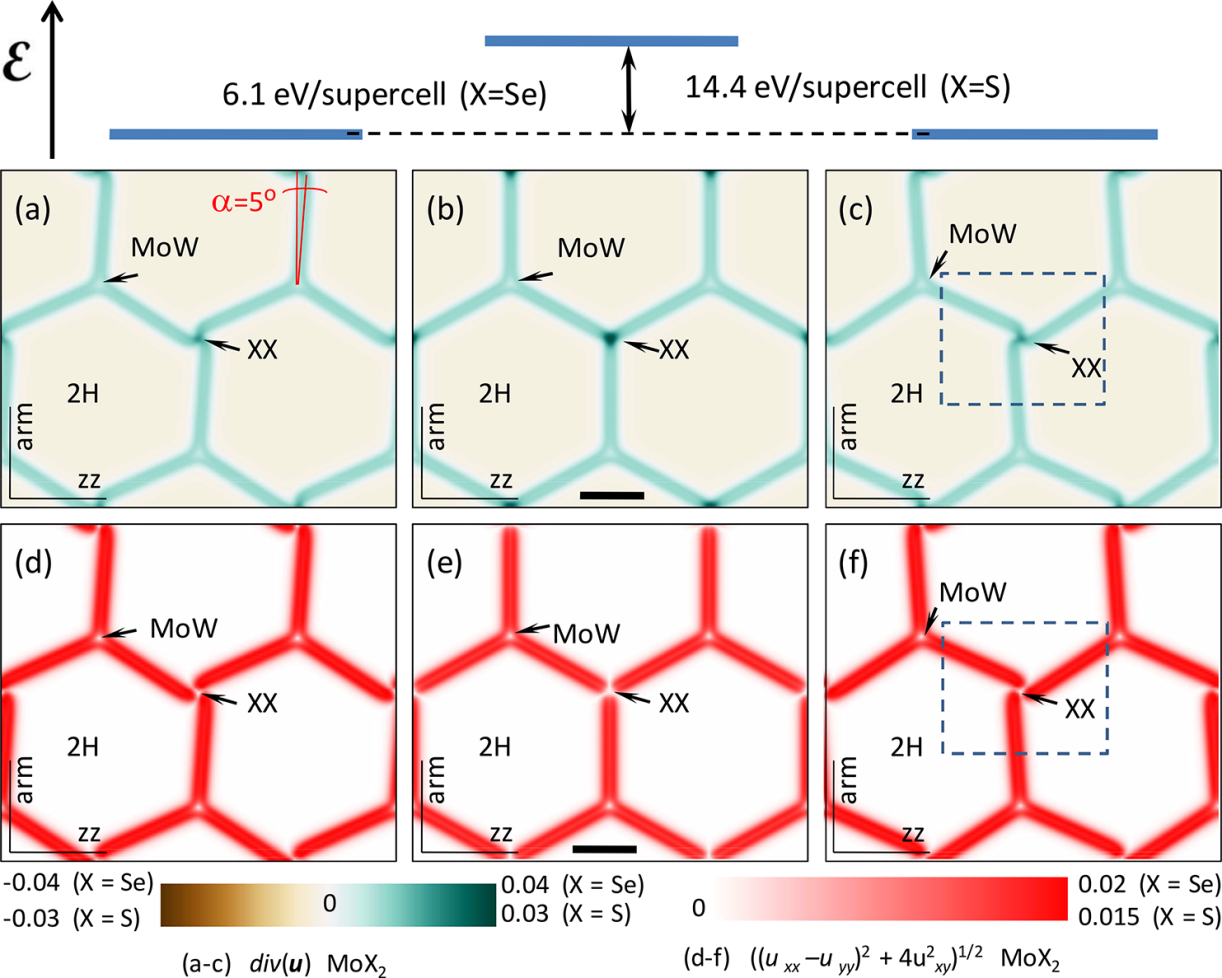
Maps of (a–c) hydrostatic and (d–f) shear components of the strain tensor in principal axes for (b) symmetric and (a and c) twirled structures of the reconstructed moiré superlattice in aligned AP-MoX_2_/WX_2_ bilayers. The top sketch shows the energy gain from the formation of twirled structures. The scale bars are 30 and 45 nm for X = Se and X = S, respectively. Dashed rectangles in panels c and f show areas displayed in Figure 5.

Lattice relaxation in AP-MoX_2_/WX_2_ heterostructures leads to the formation of hexagonal domains of 2H-like stacking (simultaneous metal-on-chalcogen and chalcogen-on-metal-like in bulk 2H TMD crystals). Those domains are separated by a hexagonal DWN with two inequivalent nodes: one with XX stacking (chalcogen on chalcogen) and the other with two metallic sites atop each other, MoW. Upon minimizing the energy in eq 3 for crystallographically aligned bilayers, we identify two candidates for the lowest-energy configuration of DWN: symmetric and left/right-handed twirled structures (Figure 4). Twirled structures form around XX nodes, whereas the DWN around MoW nodes is almost unchanged (rotated as a whole by only ≈5°), which is because those nodes host metastable MoW stacking with a much weaker hydrostatic strain component as compared to that of XX nodes. Energies of symmetric and twirled structures listed in Table 3 show that the broken-symmetry structure is energetically favorable. As in the P case, this broken-symmetry structure preserves the periodicity of the nonrelaxed moiré superlattice and substantially differs from that discussed in ref (10) for twisted TMD homobilayers.

**Table 3:** Adhesion, Elastic, and Total Energies (in electronvolts per supercell) Computed for Symmetric (sym) and Twirled (twirl) Structures and Total Energy Differences, Δ, for Aligned AP-MoX_2_/WX_2_ Bilayers

X	structure	elastic	adhesion	total	Δ
Se	sym	329.46	–2648.55	–2319.09	6.12
	twirl	323.77	–2648.98	–2325.21	
S	sym	496.25	–5904.35	–5408.10	14.44
	twirl	482.70	–5905.24	–5422.54	

**Figure 5:**
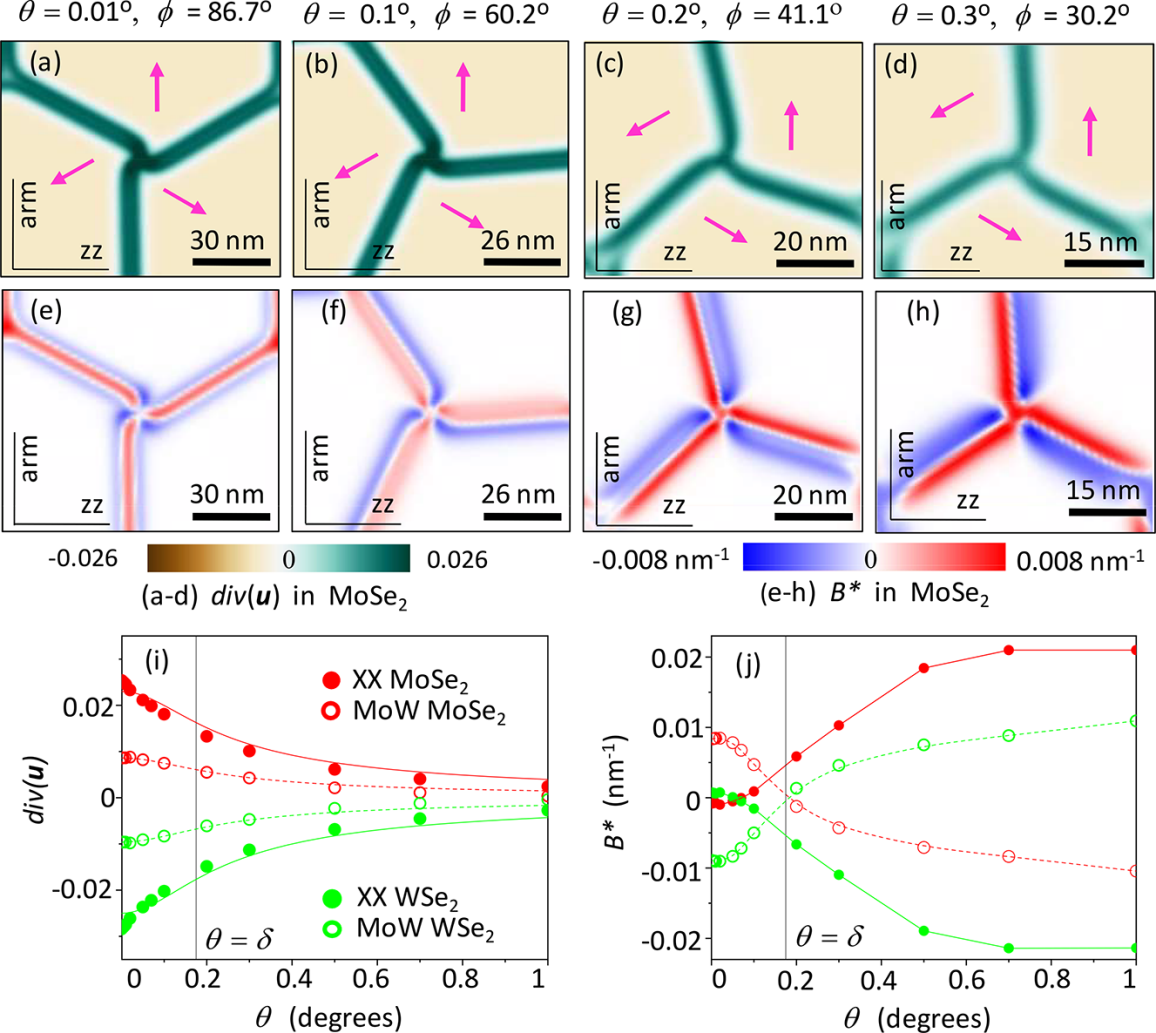
Twirl in AP-MoSe_2_/WSe_2_ for θ = 0.01°, 0.1°, 0.2°, and 0.3°. (a–d) Maps of div**u**_Mo_. The arrows indicate the direction of stacking vector **r**_0_ () inside domains. (e–h) B* = 2∂_*x*_*u*_*xy*_^Mo^ + ∂_*y*_(*u*_*xx*_^Mo^ – *u*_*yy*_^Mo^). (i) Values of div**u**_Mo,W_ at the twirl center for various twist angles. (j) B* dependence in centers of XX (twirl) and MoW nodes.

Figure 5 shows twist-angle dependences of strain field characteristics around a single twirl. The data for div**u** in Figure 5i indicate a decrease in its magnitude (in each of the layers) with the twist angle. This trend corresponds to the decay of twirling with the growth of the misorientation of the layers (Figure 5a–d) as already discussed about P heterostructures in the previous section. We also point out that, in contrast to P bilayers, for AP heterostructures shear strain results in the same signs of piezocharge densities (∝*e*_11_^Mo^*B*_Mo_^*^ ≈ *e*_11_^W^*B*_W_^*^) in both layers (opposite signs of piezocoefficients, *e*_11_^Mo^ ≈ −*e*_11_^W^, due to the antialignment of layers, are compensated by sign inversion for *B*_Mo_^*^ ≈ −*B*_W_^*^). Note that DWN nodes host an area of non-zero B*, which reverses sign at θ ≈ δ, with the magnitude at MoW nodes being substantially higher than that of twirled nodes (see Figure 5j).

## Conclusions

The main finding of this study consists of the prediction of a spontaneous symmetry breaking in the form of a domain wall network in (almost) perfectly aligned MoX_2_/WX_2_ heterostructures. This symmetry breaking consists of the formation of twirl-shaped nodes, caused by a substantial reduction in the hydrostatic strain in the vicinity of those nodes. The overall structure chooses one of the two (right- or left-handed) orientations in all nodes of the DWN, which we tested by relaxing the lattice across few-fold enlarged supercells. This distinguishes twirls in Figure 3 and Figure S7 from the moiré pattern asymmetry in TMD homobilayers; random twisting of DWN nodes across the network was discussed in ref (10). We also note that aperiodic twirling was predicted in marginally twisted bilayer graphene,^9^ where it was expected to be accompanied by bulging of the bilayers by ≤2 Å. The difference between TMDs and graphitic systems is determined by the much higher rigidity of carbon monolayers. Moreover, encapsulation of bilayers, graphene or TMDs, between hBN crystals would prohibit bulging, suppressing twirling as discussed in ref (9) in contrast to the symmetry-breaking transition discussed here for TMD heterobilayers, where encapsulation into an incommensurate environment would not suppress twirling (as the latter does not involve substantial out-of-plane displacements).

The reduction of the hydrostatic strain component by twirling could have a pronounced effect on the energetics of the band edge states of the electrons and holes, making those less confined at the twirled nodes, as compared to symmetric nodes. As a result, the earlier studies^11^ overestimated electron, hole, and exciton binding at the XX DWN nodes. Because twirling reduces the maximal hydrostatic strain at XX nodes from ∼6% to ∼2%, we expect much smaller band edge shifts for both electrons and holes, and at least 2 times smaller binding energies for charge carrier and interlayer excitons as compared to those in ref (11) , which requires further studies beyond the scope of this paper.

## Supporting Information Available

Mutual orientation of the crystalline and moire superlattice, lateral offset *r*_0_ for P- and AP-oriented bilayers, dependence of energy gain Δ on twist angle and convergence of results with respect to the grid interval, curl of displacement for symmetry-broken states, and the adhesion energy formula (PDF)
