# Supplementary material for: Twirling and Spontaneous Symmetry Breaking of Domain Wall Networks in Lattice-Reconstructed Heterostructures of Two-Dimensional Materials
Source: Nano Lett. 2023 Oct 2;23(19):8875–80. doi: 10.1021/acs.nanolett.3c01896 (PMC10571146; doi:10.1021/acs.nanolett.3c01896)
Supplement: Supplementary file 1 — nl3c01896_si_001.pdf [file nl3c01896_si_001.pdf]

# Supporting Information for "Twirling and spontaneous symmetry breaking of domain wall networks in lattice-reconstructed heterostructures of 2D materials"

Mikhail A. Kaliteevski,† Vladimir Enaldiev,† and Vladimir I. Fal'ko*,†

†National Graphene Institute, University of Manchester, Booth St. E., Manchester M13 9PL, United Kingdom

‡Department of Physics and Astronomy, University of Manchester, Manchester M13 9PL, United Kingdom

¶Henry Royce Institute for Advanced Materials, Manchester, M13 9PL, United Kingdom

E-mail: vladimir.falko@manchester.ac.uk

## Mutual orientation of crystalline and moire superlattice.

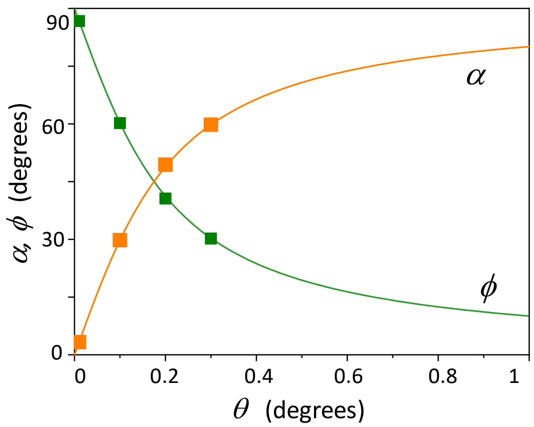


Figure S1: Dependence of angle $\alpha$ between zigzag direction and moire lattice vector (orange) and angle $\phi$ between armchair direction moire lattice vector (green) on twist angle $\theta$. Symbols correspond to the angles shown in figures 4 and 5 of the main paper.

For twist angle $\theta$=0, direction of moire superlattice vector corresponds to zig-zag direction of crystalline lattice (and coincides with orientation of the domain wall of symmetric state for P-orientation). The nearest armchair direction (which could be parallel or anti-parallel to crystalline lattice basic vector) is rotated by $\pm 30°$ due to $C_3$-symmetry of TMD. With increase of twist angles moire superlattice rotates with respect to crystalline lattice and angle $\alpha$ between zigzag direction and moire lattice vector reads $\alpha = \pi/3 - \arctan\left[(\sqrt{3}\delta - \theta)/(\sqrt{3}\theta + \delta)\right]$, $\alpha$=$\pi/2-\phi$. Here $\phi$ is an angle between armchair direction and moire lattice vector as shown in figure S1. for homobilayers (when $\delta = 0$), the domain wall is oriented along zigzag direction for P-oriented bilayer and along armchair direction for AP-oriented bilayer.

The vector $\mathbf{A}$ defining pseudomagnetic field $\mathbf{B}^*$, in the frame coupled coupled to crystalline lattice (where x-direction is parallel zig-zag) reads

$\mathbf{A} = \left[\frac{\partial u_x}{\partial x} - \frac{\partial u_y}{\partial y}, \ -\frac{\partial u_x}{\partial y} - \frac{\partial u_y}{\partial x}\right]$, while the expression for $\mathbf{A}$ in the frame, coupled to the moire lattice has a form

$$\mathbf{A} = \begin{bmatrix} \cos(3\alpha) & \sin(3\alpha) \\ -\sin(3\alpha) & \cos(3\alpha) \end{bmatrix} \begin{bmatrix} \frac{\partial u'_{x'}}{\partial x'} - \frac{\partial u'_{y'}}{\partial y'} \\ -\frac{\partial u'_{x'}}{\partial y'} - \frac{\partial u'_{y'}}{\partial x'} \end{bmatrix}. \tag{S1}$$

.

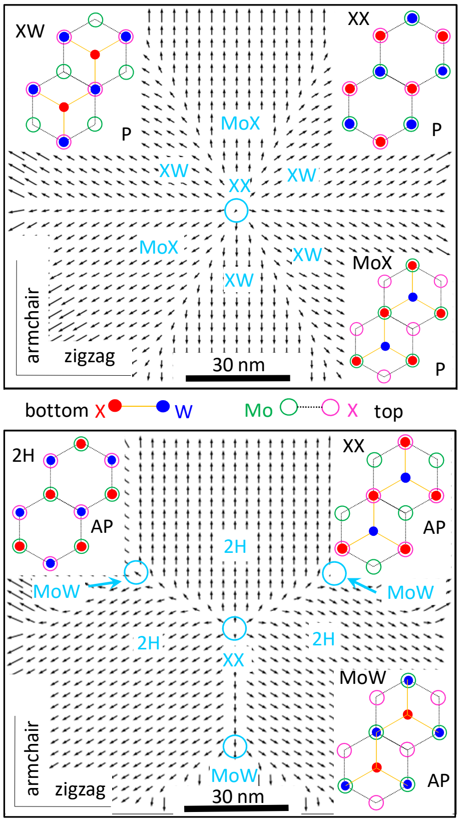


Figure S2: Map of vector $r_0$ (shown not to scale) $MoSe_2$-layer for symmetric state for P-oriented bilayers $MoSe_2$ (top panel) and for AP-oriented bilayers (bottom layers), shown not to scale. Inset show layout of atoms in symmtry points and domains

.

## Lateral offset $r_0$ for P- and AP- oriented bilayers.

Density of adhesion energy defined by eq. 1 in the main manuscript are defined by lateral offset vector $\mathbf{r_0}$, shown in figure S2.

It can be seen that withing the domains vector $\mathbf{r_0}$ is oriented along armchair direction and corresponds to half of the distance between opposite nodes of the hexagone, formining cryslalline lattice: $r_0 \approx a/\sqrt{3}$. For P-oriented bilayers domain wall is oriented along zig-zag direction, while for AP-orientation domain wall are oriented along armchair direction. The same orientation of the $r_0$ within the domains occures in the case of twirled domain wall as shown in figures 3 and 5 of the main manuscript.

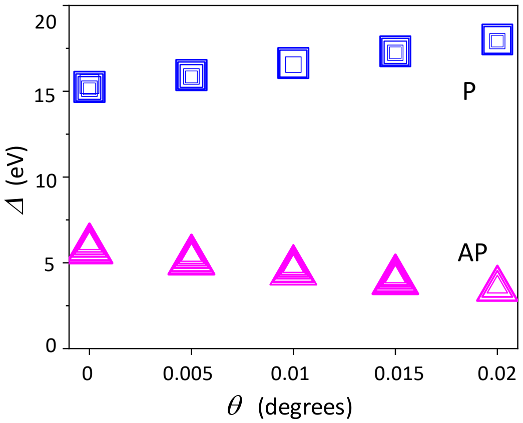


Figure S3: Dependence of energy gain between symmetric and broken-symmetric states for P- (blue squares) AP- magenta triangles on the twist angle $\theta$. Modeling was performed using grid interval 1 nm, 1.1 nm, 1.2 nm, 1.4 nm, and 1.5 nm (shown here by symbols of different sizes).

## Dependence of energy gain $\Delta$ on twist angle and convergence of results in respect to grid interval.

Since the results are obtained by numerical modeling using a grid with finite interval, the convergence of the results in respect to the variation of the grid interval should be demonstrated.

The formalism used is based on the analysis of the system as contineous media, but the lattice parameters of TMG is about 0.3 nm, is included into expression for adhesion energy (eq. 1 of the main paper) . Figure S3 shows the dependence of $\Delta$ on twist calculated using several grid interval from 1 nm to 1.5 nm: it can be seen, that convergence is achieved. When grid interval goes beyond 2 nm, the calculated value of $\Delta$ start to depend of the grid interval.

It is interesting to note that for P- (AP-) orientation the gain $\Delta$ is increasing (decreasing)

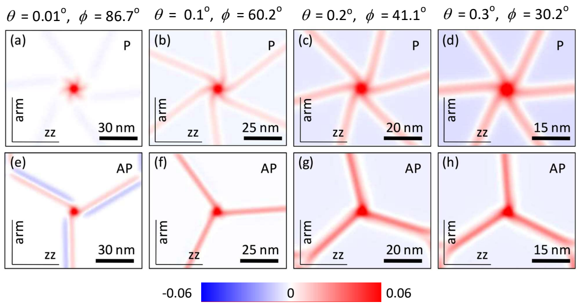


Figure S4: Maps of $rot(\mathbf{u})$ in $MoSe_2$ layer for P - (a,b,c,d) and AP - (e,f,g,h) oriented bilayers for twist angles $\theta = 0.01°$ (a,e), 0.1 ° (b,f), 0.2° (c,g), and 0.3° (d,h).

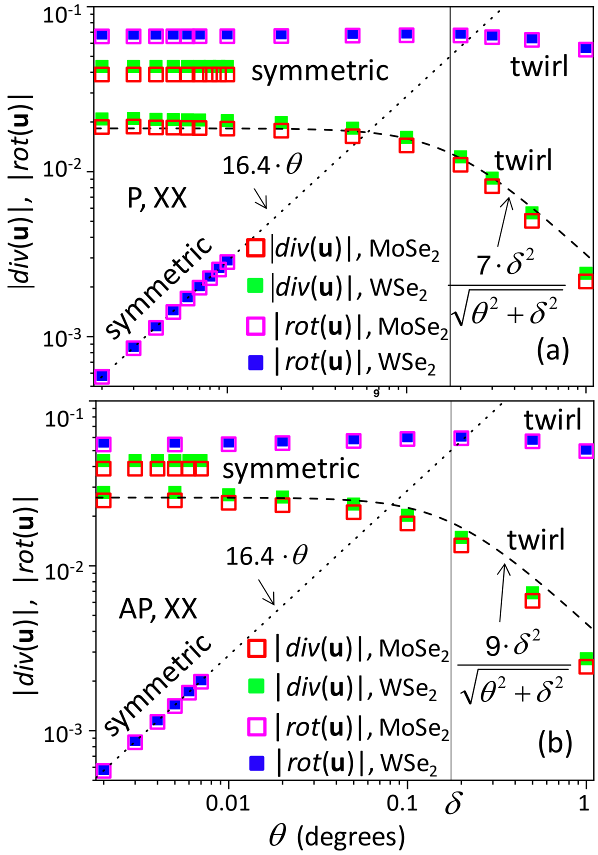


Figure S5: Dependence on the twist angle $\theta$ of the magnitudes of curl and divergence of the displacement at the XX point for P-oriented bilayers (a) and AP-oriented (b) for $MoSe_2$ and for $WSe_2$.

with increasing twist angle $\theta$.

## The curl of displacement for symmetry-broken states

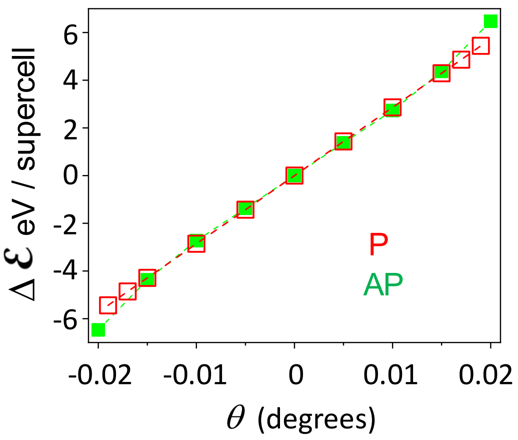


Figure S6: Total energy difference, $\Delta\mathcal{E} = \mathcal{E}_L - \mathcal{E}_R$, between left-handed and right-handed twirled structures as a function of a twist angle $\theta$.

For the analysis of the properties of the domian structure it is useful to analyse a behaviour of the curl of displacement $rot(\mathbf{u})$. For symmetric configuration and zero twist angle, at XX-point $rot(\mathbf{u}) = \mathbf{0}$ for both AP-and P - orientations. Within the domain, $rot(\mathbf{u}) = \theta$ for both P- and AP -orientations and for both symmetric configuration, as illustrated in figure S4.

For twirled configuration there is a pronounced maximum of $rot(\mathbf{u})$ at XX-point (see figure S4) and the value of rot(u) does not change substantially with increasing twist angle $\theta$, as shown in figure S5. On other hand for symmetric state magnitude of $rot(\mathbf{u})$ at XX - point growth linearly with increasing $\theta$, and can be described by approximate formula $rot(\mathbf{u}) = 16.4\theta$. It is interesting to note that $rot(\mathbf{u})$ for symmetric configuration interpolated to larger $\theta$ become equal to $rot(\mathbf{u})$ for twirled configuration when $\theta = \delta$. Thus, for twist angle $\theta > \delta$ , the difference between symmetric configuration disappears, and domain walls become straight lines.

For finite $\theta$ the total energy of left- and right-handed twirled structures is different as shown in Figure S6. The more energetically favorable becomes twirled structure for which direction of twirls in XX nodes and interlayer $\theta$-twist coincide. The total energy difference scales linearly at the small $\theta$, deviating from the linear dependence for larger $\theta$ due to shrinkage of moiré cell area.

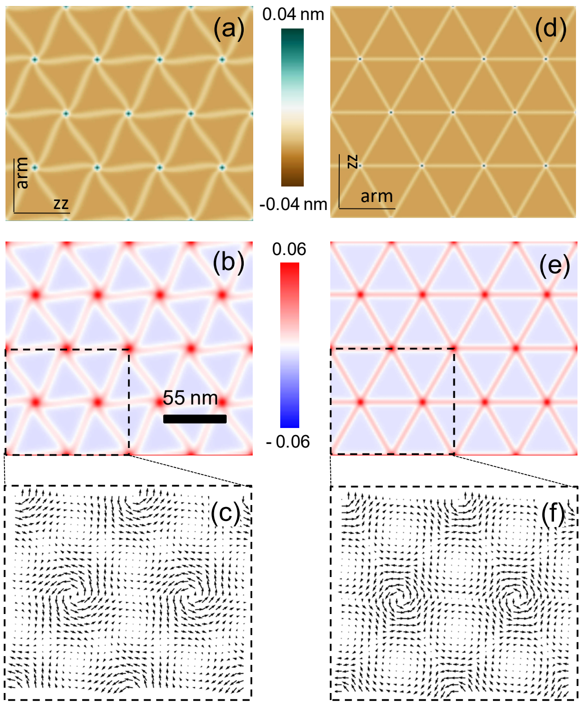


Figure S7: Variation of interlayer distance (a,d), curl of displacement (b,e) in $MoSe_2$ obtained using 2x2 supercell (highlighted by rectangular) for $\theta = 0.3°$. The vectors of displacement are shown in figure (c,f). Figures (a,b,c) correspond to heterobilayers $MoSe_2$/ $WSe_2$, while figures (d,e,f) corresponds to homobylayers $MoSe_2$/ $MoSe_2$.

# Adhesion energy formula

In Ref.[S1,S2] we established the following equation for adhesion energy of P/AP-$MoX_2$/$WX_2$ heterostructures:

$$W_{P/AP}(\boldsymbol{r}_0) = -\varepsilon Z^2(\boldsymbol{r}_0) + w_1 \sum_{n=1,2,3} \cos\left(\boldsymbol{G}_n^{(1)} \boldsymbol{r}_0\right) + w_2 \sum_{n=1,2,3} \sin\left(\boldsymbol{G}_n^{(1)} \boldsymbol{r}_0 + \gamma_{P/AP}\right). \quad \text{(S2)}$$

$$Z(\boldsymbol{r}_0) = \frac{1}{2\varepsilon} \sum_{n=1}^{3} \left[ w_1 \sqrt{G^2 + 1/\rho^2} \cos\left(\boldsymbol{G}_n^{(1)} \boldsymbol{r}_0\right) + w_2 G \sin\left(\boldsymbol{G}_n^{(1)} \boldsymbol{r}_0 + \gamma_{P/AP}\right)\right], \quad \text{(S3)}$$

where $w_1 = A_1 e^{-d_0\sqrt{G^2+1/\rho^2}}$ and $w_2 = A_2 e^{-d_0 G}$ and parameter values are gathered in Table S1. Substitution of (S3) into (S2) and expansion of quadratic terms leads to the Eq. (1) of the main text:

$$W_P(\boldsymbol{r}_0) = \left[ w_1 + w_2 - \frac{\left(w_1\sqrt{G^2+1/\rho^2} + w_2 G\right)^2}{4\varepsilon} \right] \sum_{n=1,2,3} \cos\left(\boldsymbol{G}_n^{(1)} \boldsymbol{r}_0\right)$$
$$- \frac{\left(w_1\sqrt{G^2+1/\rho^2} + w_2 G\right)^2}{4\varepsilon} \sum_{n=1,2,3} \left[ \cos\left(\boldsymbol{G}_n^{(2)} \boldsymbol{r}_0\right) + \frac{1}{2}\cos\left(\boldsymbol{G}_n^{(3)} \boldsymbol{r}_0\right) \right];$$
(S4)

$$W_{AP}(\boldsymbol{r}_0) = \left[ w_1 - \frac{\left(w_1\sqrt{G^2+1/\rho^2}\right)^2 - (w_2 G)^2}{4\varepsilon} \right] \sum_{n=1,2,3} \cos\left(\boldsymbol{G}_n^{(1)} \boldsymbol{r}_0\right)$$
$$- \frac{\left(w_1\sqrt{G^2+1/\rho^2}\right)^2 + (w_2 G)^2}{4\varepsilon} \sum_{n=1,2,3} \cos\left(\boldsymbol{G}_n^{(2)} \boldsymbol{r}_0\right)$$
$$- \frac{\left(w_1\sqrt{G^2+1/\rho^2}\right)^2 - (w_2 G)^2}{8\varepsilon} \sum_{n=1,2,3} \cos\left(\boldsymbol{G}_n^{(3)} \boldsymbol{r}_0\right) + \left[ w_2 + \frac{w_1 w_2 G\sqrt{G^2+1/\rho^2}}{2\varepsilon} \right] \sum_{n=1,2,3} \sin\left(\boldsymbol{G}_n^{(1)} \boldsymbol{r}_0\right)$$
$$- \frac{w_1 w_2 G\sqrt{G^2+1/\rho^2}}{4\varepsilon} \sum_{n=1,2,3} \sin\left(\boldsymbol{G}_n^{(3)} \boldsymbol{r}_0\right);$$
(S5)

Table S1: Fitting parameters for adhesion energy (S2).

| | $A_1$, $eV/nm^2$ | $A_2$, $eV/nm^2$ | $\rho$, nm | $d_0$ nm | $\varepsilon$ $eV/nm^4$ |
|---|---|---|---|---|---|
| $MoS_2$/ $WS_2$ | 79160000 | 63427 | 0.0492 | 0.65 | 214 |
| $MoSe_2$/ $WSe_2$ | 77621500 | 84739 | 0.0520 | 0.69 | 189 |

# References


[S1] Enaldiev, V. V.; Zólyomi, V.; Yelgel, C.; Magorrian, S. J.; Fal'ko, V. I. Stacking Domains and Dislocation Networks in Marginally Twisted Bilayers of Transition Metal Dichalcogenides. *Phys. Rev. Lett.* **2020**, *124*, 206101.

[S2] Enaldiev, V. V.; Ferreira, F.; Magorrian, S. J.; Fal'ko, V. I. Piezoelectric networks and ferroelectric domains in twistronic superlattices in $WS_2/MoS_2$ and $WSe_2/MoSe_2$ bilayers. *2D Materials* **2021**, *8*, 025030.
